# Tick-borne haemoparasites in African buffalo (*Syncerus caffer*) from two wildlife areas in Northern Botswana

**DOI:** 10.1186/s13071-014-0627-y

**Published:** 2015-01-15

**Authors:** Dewald Eygelaar, Ferran Jori, Mokganedi Mokopasetso, Kgomotso P Sibeko, Nicola E Collins, Ilse Vorster, Milana Troskie, Marinda C Oosthuizen

**Affiliations:** Department of Veterinary Tropical Diseases, Faculty of Veterinary Science, University of Pretoria, Private Bag X04, Onderstepoort, 0110 South Africa; Department of Zoology and Entomology, University of Pretoria, Pretoria, 0002 South Africa; UPR AGIRs, CIRAD, Campus International de Baillarguet, Montpellier, 34398 France; FAO-ECTAD Office for Southern Africa, Gaborone, Botswana; Department of Animal Science and Production, Botswana College of Agriculture, Private Bag 0027, Gaborone, Botswana; Botswana Vaccine Institute, Private Bag 0031, Gabarone, Botswana

**Keywords:** Botswana, African buffalo, Haemoparasites, Tick-borne diseases, *Theileria*, *Babesia*, *Anaplasma*, *Ehrlichia*, Reverse line blot hybridization assay, Real-time PCR, IFAT

## Abstract

**Background:**

The African buffalo (*Syncerus caffer*) is a host for many pathogens known to cause economically important diseases and is often considered an important reservoir for livestock diseases. Theileriosis, heartwater, babesiosis and anaplasmosis are considered the most important tick-borne diseases of livestock in sub-Saharan Africa, resulting in extensive economic losses to livestock farmers in endemic areas. Information on the distribution of tick-borne diseases and ticks is scarce in Northern Botswana. Nevertheless, this data is necessary for targeting surveillance and control measures in livestock production at national level.

**Methods:**

In order to address this gap, we analyzed 120 blood samples from buffalo herds for the presence of common tick-borne haemoparasites causing disease in livestock, collected in two of the main wildlife areas of Northern Botswana: the Chobe National Park (CNP, n = 64) and the Okavango Delta (OD, n = 56).

**Results:**

Analysis of the reverse line blot (RLB) hybridization assay results revealed the presence of *Theileria, Babesia, Anaplasma* and *Ehrlichia* species*,* either as single or mixed infections. Among the *Theileria* spp. present, *T. parva* (60%) and *T. mutans* (37%) were the most prevalent. Other species of interest were *Anaplasma marginale* subsp. *centrale* (30%), *A. marginale* (20%), *Babesia occultans* (23%) and *Ehrlichia ruminantium* (6%). The indirect fluorescent antibody test (IFAT) indicated 74% of samples to be positive for the presence of *T. parva* antibodies. Quantitative real-time PCR (qPCR) detected the highest level of animals infected with *T. parva* (81% of the samples). The level of agreement between the tests for detection of *T. parva* positive animals was higher between qPCR and IFAT (kappa = 0.56), than between qPCR and RLB (kappa = 0.26) or the latter and IFAT (kappa = 0.15).

**Conclusions:**

This is the first report of tick-borne haemoparasites in African buffalo from northern Botswana, where animals from the CNP showed higher levels of infection than those from OD. Considering the absence of fences separating wildlife and livestock in the CNP and the higher levels of some parasite species in buffalo from that area, surveillance of tick-borne diseases in livestock at the interface in the CNP should be prioritized.

## Background

Theileriosis, babesiosis, anaplasmosis and heartwater are considered to be the most important tick-borne diseases (TBDs) of livestock in sub-Saharan Africa, resulting in extensive economic losses to farmers in endemic areas. The African buffalo (*Syncerus caffer*) is the natural reservoir host of *Theileria parva*, which is transmitted by the tick species, *Rhipicephalus appendiculatus, R. zambeziensis* and *R. duttoni* [1,2]. *T. parva* causes East Coast fever (ECF), which occurs in eastern and central Africa. ECF was introduced into southern Africa in the early 1900s through cattle importation from East Africa and was eradicated from South Africa in the 1950s [3,4]. *T. parva* also causes Corridor Disease*,* which is still prevalent in South Africa in areas where buffalo and cattle share grazing grounds in the presence of its tick vectors. It is a controlled disease in South Africa because of a concern that ECF might recur [5,6]. Although *T. parva* distributions have been described in Mozambique [7], Zambia [8] and Zimbabwe [3], no information on the distribution of *T. parva* is available for many other southern African countries, including Botswana.

In addition to *T. parva*, buffalo are also thought to be the original reservoir host of other non-pathogenic, mildly pathogenic and benign *Theileria* species namely, *Theileria mutans*, *Theileria velifera*, *Theileria buffeli*, *Theileria* sp. (buffalo) [9,10] and *Theileria* sp. (bougasvlei) [11,12]. *Theileria* parasites usually occur as mixed infections in buffalo and cattle. Although the benign and non-pathogenic forms do not have any significant economic importance, their presence could interfere with the interpretation of results obtained in some diagnostic tests designed to diagnose the pathogenic *T. parva*. Diagnostic tests for *T. parva* include microscopic examination of blood smears for the presence of piroplasms and schizonts, and serological methods such as the indirect fluorescent antibody test (IFAT) which is routinely used in South Africa for *T. parva* antibody detection in “disease free” buffalo [13,14]. Molecular diagnostic methods detect specific parasite sequences in DNA extracts from blood or tissue samples. The reverse line blot (RLB) hybridization assay makes use of polymerase chain reaction (PCR) amplification of haemoparasite small subunit ribosomal RNA genes (srRNA) which are screened with group- and species-specific probes for the simultaneous detection and identification of haemoparasites in mixed infections [15]. To date, the most sensitive molecular test for the detection of *T. parva* is a quantitative real-time PCR (qPCR) using hybridization probe chemistry, where the central region of the parasite 18S rRNA gene is amplified and the presence of *T. parva* is confirmed by melting curve analysis [16]. It is currently routinely used to test for *T. parva* infections in buffalo and cattle in South Africa as part of the Corridor disease control strategy.

It has been shown that African buffalo are also carriers of a number of other tick-borne parasites which are detrimental to livestock including *Ehrlichia ruminantium*, *Babesia bigemina*, *B. bovis*, *Anaplasma marginale* and *A. marginale* subsp. *centrale* [17-19]. Although buffalo show no disease symptoms, as reservoir hosts, they may represent a threat to the livestock industry. *Ehrlichia ruminantium*, an intracellular rickettsial bacterium, is the causative agent of heartwater (cowdriosis) and is transmitted by three-host ticks belonging to the genus, *Amblyomma* [20]*. Babesia bigemina* and *B. bovis* cause bovine babesiosis, commonly known as redwater fever. Tick vectors for these parasites include *Rhipicephalus microplus* (formerly *Boophilus microplus*) and *Rhipicephalus annulatus* (formerly *Boophilus annulatus*). It is believed that *Babesia* is the second most common blood parasite after trypanosomes representing a significant health risk for cattle [21]. *Anaplasma marginale* causes bovine anaplasmosis which is characterized by the infiltration of the host’s red blood cells. It can be transmitted to other hosts through mechanical transmission but the most important mode of transmission is via tick bites, the main tick vector being *R. decoloratus* (formerly *Boophilus decoloratus*) [22,23]. *Anaplasma marginale* subsp. *centrale* causes a milder form of anaplasmosis, and is used in a live blood vaccine in many countries, including South Africa [24].

Generally speaking, publications on significant tick-borne haemoparasites in Botswana are limited [25-28] and there are no published reports of the presence of *T. parva* in livestock in this country. In addition, published literature on the occurrence of pathogens in buffalo populations from Botswana is very scarce [29]. Therefore, the goal of this study was to determine the prevalence of tick-borne parasites circulating in two distinct buffalo populations from Northern Botswana using different diagnostic methods and to use these data to compare the performance of those tests in detecting *T. parva* in buffalo.

## Methods

### Buffalo sampling

**Figure 1:**
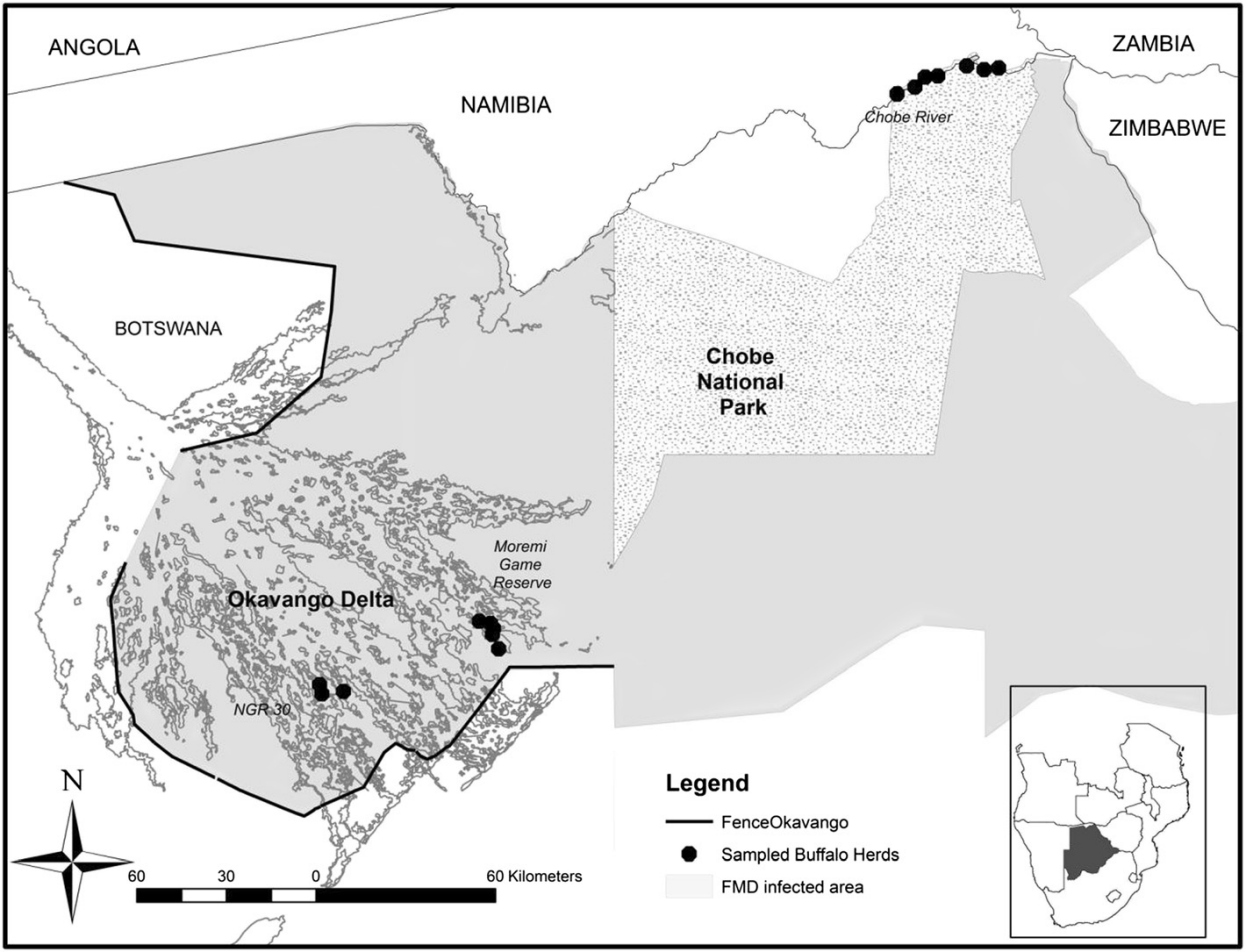
**Areas where samples were collected from buffalo herds in northern Botswana.** The three distinct areas where buffalo captures took place are indicated in italic characters. Individual capture sites are indicated by black dots.

The Chobe National Park (CNP) and Okavango Delta (OD) are located in two different districts of Northern Botswana (Chobe and Ngamiland Districts, respectively) and represent the largest wildlife areas in this part of the country. They are both integrated in the Foot and Mouth Disease infected area, a large part of the northern region of Botswana devoted to wildlife conservation in which buffalo populations are separated from the primary cattle export and buffer zones by the use of veterinary cordon fences (Figure 1). The Chobe, Zambezi and Okavango rivers are the largest in the region, providing abundant water throughout the year. Rainfall is strongly seasonal, occurring mostly from December to April (wet season). Vegetation consists mainly of deciduous dry woodland and scattered grasslands. Wildlife abundance is fundamentally dependent on rainfall and water availability and varies cyclically throughout the years [30]. The CNP encompasses 10 700 km^2^ of savannah grassland. The boundaries of CNP are natural, the Chobe river in the north constituting the natural border between Botswana and Namibia. There is no physical barrier preventing contacts between cattle and wildlife and the main water source for the animals in that area is the Chobe river which is exposed to seasonal variations of water levels. The OD encompasses 16 000 km^2^ and contrary to the CNP, it is delineated from livestock areas by a double veterinary cordon fence to prevent contacts between wildlife and cattle [29], and it is largely flooded throughout the year. According to the last available wildlife census from Northern Botswana, buffalo populations and densities are estimated at 31 500 individuals and 0.94 individuals/km^2^ in OD and 7 500 individuals and 0.23 individuals/km^2^ in the CNP [31].

The sampling process was opportunistic and details of the capture approach were described [29]. During the capture process, blood samples were collected from a total of 120 individual buffalo. In the CNP, buffalo were captured along the Chobe river and blood samples were collected from 64 individual buffalo belonging to seven distinct herds. In the OD, 8 buffalo herds were sampled in two different management units: the Moremi Game Reserve (MGR) (n = 18 individuals, 4 herds) and in the NG30 area (n = 38 individuals, 4 herds) (Figure 1). Buffalo densities in those locations were estimated at 1.88 buffalo/km^2^ for the Chobe river, 1.37 buffalo/km^2^ for the MGR and 3.55 buffalo/km^2^ for the NG30 area of the OD (31). The sex and age of the animals were recorded. Age was measured according to dentition; animals younger than 3 years were considered as young and animals older than 3 years were considered to be adults. Whole blood samples were obtained from the jugular vein, maintained in refrigeration and sent to the Botswana National Veterinary Laboratory in Gaborone. There, they were centrifuged at 1500 xg for 15 minutes. Sera was then harvested with a pipette and stored frozen at −20°C, until the samples were ready to be sent to the Agricultural Research Council–Onderstepoort Veterinary Institute (ARC-OVI) in South Africa for analysis.

### Blood smears and DNA extraction

A total of 120 thin layer smears were prepared in the field from a blood drop of the ear sublime vein of the captured buffalo (two individuals were missed). Those were dried in the sun and fixed with methanol. At the laboratory, each smear was stained with Giemsa dye following the standard procedures. Genomic DNA was extracted from the Giemsa-stained slides using the QIAmp DNA mini kit (Qiagen) following the manufacturer’s protocol. The DNA was eluted into 100 μl of TE buffer and stored at −20°C until further use.

### PCR amplification and reverse line blot (RLB) hybridization assay

A total of 120 DNA samples were tested using the RLB hybridization assay as previously described [15,7]. Briefly, the V4 hypervariable region of the parasite 18S rRNA gene was amplified using primers RLB-F2 and RLB-R2 [32], while the V1 region of the parasite 16S rRNA gene was amplified from *Ehrlichia* and *Anaplasma* species using primers Ehr-F and Ehr-R [33]. The PCR reaction was prepared as follows: 5 μl DNA (30–50 ng), 12.5 μl Platinum Quantitative PCR SuperMix-UDG (Invitrogen, The Scientific Group, South Africa), and 20 pmol of each primer made up to a total volume of 25 μl using nuclease-free water. Amplification was done using a touchdown PCR programme as previously described [32]. A *T. parva* positive buffalo DNA sample, 102 [16], and nuclease-free water were used as positive and negative controls, respectively. Amplicons were visualised on a 2% ethidium bromide-stained agarose gel and then screened by the RLB hybridization assay as previously described [15,34]. The *Theileria*, *Ehrlichia*, *Anaplasma* and *Babesia* group- and species-specific oligonucleotide probes used were the same as those described in [35] and [36].

### Indirect fluorescent antibody test (IFAT)

After discarding haemolysed samples, only a total of 108 serum samples collected from buffalo were available to be tested using the IFAT [13,37,38] according to OIE standards [39]. The test was conducted at the ARC-OVI using two dilutions, 1/40 and 1/80. The presence of fluorescence in both the 1/40 and 1/80 dilutions was considered as a positive result in serum from buffalo, indicative of the presence of *T. parva* antibodies (Olivier Matthee, personal communication).

### T. parva*-specific quantitative real-time PCR (qPCR)*

Among the 120 smears collected, one sample was discarded because of an insufficient amount of DNA for the test. A total of 119 DNA samples were subjected to the *T. parva-*specific qPCR assay as previously described [16]. Briefly, the *T. parva*-specific forward and *Theileria* genus-specific reverse primers [16] were used to amplify a 167 bp fragment of the parasite V4 variable region of the 18S rRNA gene. For the specific detection of *T. parva* amplicons, the hybridization probes *T. parva* anchor and *T. parva* sensor (640 nm LC Red) were included in the PCR reaction which consisted of 4 μl of 10× LightCycler-FastStart DNA Master^PLUS^ Hybridization Probes mix (with 2× final concentration), 0.5 mM of each primer, 0.1 mM of each hybridization probe, 0.5 U Uracil-deoxy-glycosylase (UDG) and 4 μl of the template DNA (30–50 ng) with a final volume of 20 μl. A *T. parva* positive buffalo DNA sample, 102 [16], and nuclease-free water were used as positive and negative controls, respectively. Amplification and melting curve analysis were done as previously described [16] in a LightCycler1 v2 (Roche Diagnostics, Mannheim, Germany). Fluorescence values were measured at 640 nm.

### Statistical analysis

Descriptive epidemiological measures were analyzed using Epi-Info software (CDC, Atlanta, USA) and were reported as percentages of positive animals to the different diagnostic tests. Chi square test calculations for homogeneity of two populations (Fischer exact test) were used to statistically evaluate the potential influence of age, sex, location and density of buffalo at the capture sites on the observed parasite prevalence. When the variance of the two groups was not homogenous, the Kruskall Wallis test was used. Values of p < 0.05 were considered significant. Agreement between the different diagnostic tests assessing the presence of *T. parva* or its antibodies (IFAT, qPCR and RLB) was calculated for those sera having a common result to those tests. Two by two comparisons of the results were expressed using the kappa value. Kappa is a widely used measure of test agreement defined as the quotient of the observed proportion of agreement beyond chance and the maximal proportion of agreement beyond chance [40]. A kappa of 0 indicates no agreement beyond chance, while a kappa of 1 indicates perfect agreement. A kappa of 0.5 indicates a moderate level of agreement.

### Ethical statement

The study (Project nr. V082-12) was approved by the University of Pretoria Animal Ethics committee.

## Results

### RLB

The RLB results (Table 1) indicated the presence of *Theileria*, *Babesia*, *Anaplasma* and *Ehrlichia* species, either as single or as mixed infections in the buffalo populations from two wildlife areas assessed in northern Botswana. From a total of 120 blood smear samples tested, 23 samples (19.2%) contained single infections while 80 (66.7%) contained mixed infections. The most prevalent haemoparasite in the CNP was *T. mutans* (60.9%) followed by *T. parva* (51.6%), *T. buffeli* (46.9%), *B. occultans* (40.6%) and *A. marginale* subsp. *centrale* (31.3%). In the OD, *T. parva* (69.6%) was most prevalent followed by *A. marginale* subsp. *centrale* (28.6%) and *Theileria* sp. (buffalo) (23.2%).

**Table 1:** **The occurrence of different haemoparasites in buffalo blood samples from two geographical areas in northern Botswana as determined by the RLB hybridization assay**

	**Chobe National Park (n = 64)**	**Okavango delta (n = 56)**	**Total (n = 120)**
**Single infections:**	**5 (7.8%)**	**18 (32.1%)**	**23 (19.2%)**
*T. parva*	1 (1.6%)	15 (26.8%)	16 (13.3%)
*T. mutans*	3 (4.7%)	0	3 (2.5%)
*A. marginale* subsp. *centrale*	0	2 (3.6%)	2 (1.7%)
*A. marginale*	1 (1.6%)	1 (1.8%)	2 (1.7%)
**Mixed infections**	**53 (82.8%)**	**27 (48.2%)**	**80 (66.7%)**
*T. parva*	32 (50.0%)	24 (42.9%)	56 (46.6%)
*T. mutans*	36 (56.3%)	5 (8.9%)	41 (34.2%)
*A. marginale* subsp. *centrale*	20 (31.3%)	14(25.0%)	34 (28.3%)
*T. buffeli*	30 (46.9%)	4 (7.1%)	34 (28.3%)
*B. occultans*	26 (40.6%)	2 (3.6%)	28 (23.3%)
*Theileria* sp*.* (sable)	25 (39.1%)	2 (3.6%)	27 (22.5%)
*A. marginale*	13 (20.3%)	9 (16.1%)	22 (18.3%)
*Theileria* sp. (buffalo)	8 (12.5%)	13 (23.2%)	21 (17.5%)
*T. velifera*	9 (14.1%)	1 (1.8%)	10 (8.3%)
*E. ruminantium*	4 (6.3%)	3 (5.4%)	7 (5.8%)
*T. ovis*	3 (4.7%)	1 (1.8%)	4 (3.3%)
*Anaplasma* sp. Omatjenne	1 (1.6%)	2 (3.6%)	3 (2.5%)
*B. bovis*	0	2 (3.6%)	2 (1.7%)
***Theileria/Babesia*** **genus-specific only**	**2 (3.1%)**	**5 (8.9%)**	**7 (5.8%)**
***Anaplasma/Ehrlichia*** **genus-specific only**	**1 (1.6%)**	**3 (5.4%)**	**4 (3.3%)**
**Negative/below detection limit**	**2 (3.1%)**	**1 (1.8%)**	**3 (2.5%)**

RLB results indicated that a total of 72 of the 120 samples (60.0%) tested positive for *T. parva* DNA. There was a significant difference (p = 0.042) in the prevalence of *T. parva*-positive samples between the two wildlife areas. However, there was no significant association between prevalence of *T. parva,* and sex or age of the sampled animals (Table 2).

**Table 2:** **Comparison of prevalence of** ***T. parva*** **per location, age and sex with the three different tests performed**

**Parameter**		**RLB**	**IFAT**	**qPCR**
**Wildlife area**	CNP	**33/64 (51.6%)**	**39/60 (65.0%)**	49/63 (77.8%)
	OD	**39/56 (69.6%)**	**41/48 (85.4%)**	47/56 (83.9%)
**Age**	Young	19/38 (50.0%)	26/36 (72.2%)	27/38 (71.1%)
	Adult	48/77 (62.3%)	54/71 (76.1%)	66/77 (85.7%)
**Sex**	Male	23/45 (51.1%)	31/43 (72.1%)	37/45 (82.2%)
	Female	44/70 (62.9%)	49/64 (76.6%)	57/70 (81.4%)

Boldfaced values indicate a significant difference between test results for a given parameter (p ≤ 0.05).

RLB results also showed significant differences in the prevalence of *T. mutans*, *T. buffeli*, *B. occultans*, *Theileria* sp. (sable) and *T. velifera* infections per wildlife area (Table 3). There was a significant difference (p ≤ 0.05) between the age of animals that harboured *A. marginale* subsp. *centrale*, *T. buffeli*, *B. occultans* and *A. marginale* infections (Table 3). There was a significant association (p ≤ 0.05) between sex and the buffalo that tested positive for *A. marginale* subsp. *centrale* and *T. velifera* DNA (Table 3).

**Table 3:** **Comparison of occurrence of other haemoparasites per wildlife area, sex and age as determined by the RLB hybridization assay**

		***T. mutans***	***A*** **.** ***marginale*** **ss** ***centrale***	***T. buffeli***	***B. occultans***	***Theileria*** **sp. (sable)**	***A. marginale***	***Theileria.*** **sp (buffalo)**	***T. velifera***	***E. ruminantium***	***T. ovis***	***Anaplasma*** **sp. Omatjenne**	***B. bovis***
**Area**	CNP	**39/64 (60.9%)**	20/64 (31.3%)	**30/64 (46.9%)**	**26/64 (40.6%)**	**25/64 (39.1%)**	14/64 (21.9%)	8/64 (12.5%)	**9/64 (14.1%)**	4/64 (6.3%)	3/64 (4.7%)	1/64 (1.6%)	0/64 (0.0%)
	OD	**5/56 (8.9%)**	16/56 (28.6%)	**4/56 (7.1%)**	**2/56 (3.6%)**	**2/56 (3.6%)**	10/56 (17.9%)	13/56 (23.2%)	**1/56 (5.4%)**	3/56 (5.4%)	1/56 (1.8%)	2/56 (3.6%)	2/56 (3.6%)
**Age**	Young	16/38 (42.1%)	19/38 (50.0%)	**6/38 (15.8%)**	**4/38 (10.5%)**	7/38 (18.4%)	**15/38 (39.5%)**	4/38 (10.5%)	2/38 (5.3%)	2/38 (5.3%)	2/38 (5.3%)	0/38 (0.0%)	1/38 (2.6%)
	Adult	27/77 (35.1%)	16/77 (20.8%)	**27/77 (35.1%)**	**24/77 (31.2%)**	20/77 (26.0%)	**8/77 (10.4%)**	15/77 (19.5%)	8/77 (10.4%)	5/77 (6.5%)	2/77 (2.6%)	3/77 (3.9%)	0/77 (0.0%)
**Sex**	Male	21/45 (46.7%)	19/45 (42.2%)	16/45 (35.6%)	14/45 (31.1%)	13/45 (28.9%)	13/45 (28.9%)	10/45 (22.2%)	**7/45 (15.6%)**	4/45 (8.9%)	3/45 (6.7%)	1/45 (2.2%)	1/45 (2.2%)
	Female	22/70 (31.4%)	16/70 (22.9%)	17/70 (24.3%)	14/70 (20.0%)	14/70 (20.0%)	10/70 (14.3%)	9/70 (12.9%)	**3/70 (4.3%)**	3/70 (4.3%)	1/70 (1.4%)	2/70 (2.9%)	0/70 (0.0%)

Boldfaced values indicate a significant difference between test results for a given parameter (p ≤ 0.05).

**Figure 2:**
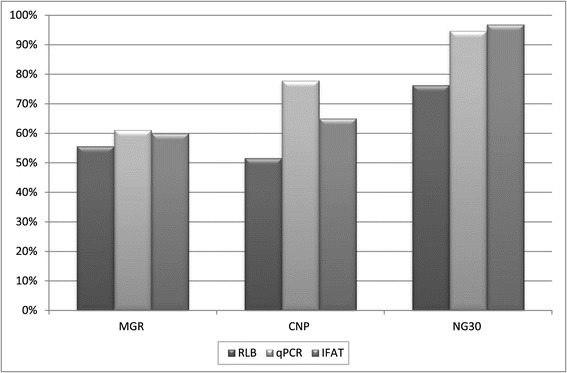
**Pair wise comparison of capture location and prevalence of** ***T. parva*** **according to the different tests performed.** Differences were significant (p ≤ 0.05) when comparing CNP and NG30 (for the 3 tests) and when comparing MGR and NG30 (for IFAT and qPCR). Note: buffalo densities in those locations were estimated at 1.88 buffalo/km^2^ for the Chobe river, 1.37 buffalo/km^2^ for the MGR and 3.55 buffalo/km^2^ for the NG30 area of the OD.

When comparing the *T. parva* RLB results in the three buffalo capture sites, *T. parva* prevalence was highest in the NG30 samples (76.3%) followed by the MGR (55.6%) samples and the CNP samples (51.6%). Differences in *T. parva* prevalence as determined by RLB were only significant when comparing NG30 versus CNP (p = 0.01) (Figure 2). There were no significant differences between the prevalence of other haemoparasites in the different capture sites or the numbers were too small to assess statistical differences

### IFAT

*T. parva* antibodies were detected in 80 of the 108 (74.1%) samples tested (Table 2). Of these, 40 (37.0%) samples tested positive at 1/80 and 40 (37.0%) at 1/40. More buffalo from OD (85.4%) than from CNP (65.0%) were seropositive for *T. parva* (Table 2) and this difference was significant (p = 0.016). There was no significant difference between the age and sex of the animals that were seropositive (Table 2). Mean seroprevalence was higher (96.9%) in the NG30 region than in the other two capture sites, 65.0% in the CNP and 60.0% in the MGR and those differences were highly significant. Differences in *T. parva* seroprevalence between MGR and CNP were not significant (Figure 2).

### Quantitative real-time PCR (qPCR)

**Figure 3:**
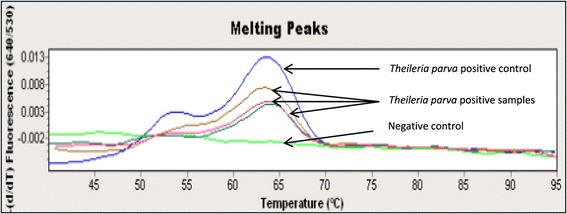
**Representative melting curves at ±63°C at 640 nm confirming the presence of** ***T. parva*** **positive samples.**

Melting curve analysis (Figure 3) confirmed the presence of *T. parva* DNA in 96 of 119 (80.7%) samples tested. There were no significant differences between prevalence of *T. parva* with this test, and the wildlife area, age or sex of the animals (Table 2), but some significant differences were found when comparing capture sites. Mean prevalence of *T. parva* as determined by qPCR was higher in the NG30 region (94.7%) than in the other two capture sites; 77.8% in the CNP and 61.1% in the MGR, respectively (Figure 2). The differences in *T. parva* prevalence between MGR and the CNP were not significant but it became highly significant when comparing *T. parva* prevalence in NG30 with that in the other two areas.

### Comparison of tests

For IFAT, 80/108 (74.1%) samples tested positive for *T. parva* antibodies, while for the qPCR and the RLB hybridization assay, 96/119 (80.7%) and 72/120 (60.0%) of the samples tested positive, respectively. The Kappa value when comparing IFAT and qPCR indicated a moderate level of agreement (0.561), while the comparison with RLB and the two other tests indicated a low level of agreement (Table 4). The observed prevalences of *T. parva* (RLB assay) and *T. parva* antibodies (IFAT) were both significantly higher in the OD than in CNP, while no significant difference was observed for the qPCR test.

**Table 4:** **Agreement expressed by kappa value when comparing diagnostic tests for** ***Theileria parva*** **two by two**

**Tests compared**	**Sample size**	**Kappa value**	**Standard error 95% CI**
**RLB vs qPCR**	119	0.256	0.09 [0.09;0.472]
**IFAT vs qPCR**	107	0.561	0.096 [0.3;0.7]
**IFAT vs RLB**	107	0.154	0.094 [−0.031;0.3]

## Discussion

This is the first report on the presence of tick-borne haemoparasites in African buffalo from two of the most representative wildlife areas from northern Botswana. Our results provide new insights on the distribution of haemoparasites from buffalo transmissible to cattle across the two main wildlife areas in northern Botswana which can be inferred to the distribution of their specific vectors. Several important pathogenic haemoparasites which could present a constraint to the livestock industry in Botswana were identified. These included *T. parva*, *A. marginale*, *B. bovis* and *E. ruminantium*. A higher level of mixed infections was found in CNP compared to the locations sampled in the OD. This may be due to the fact that the Chobe river is the main source of water in the CNP and a high number of buffalo herds congregate along this water source during the dry season which might facilitate the exchange of ticks and their parasites between different individuals. In the OD, there is water all year round and despite some animal densities might be locally higher, buffalo herds might have less interactions between each other.

The RLB, IFAT and qPCR tests all indicated a high prevalence of *T. parva* presence or exposure in both CNP and OD. This indicates a high risk of spreading Corridor disease caused by *T. parva* from buffalo to cattle by the vector ticks at the livestock-wildlife interface. Other haemoparasites with high prevalence identified by the RLB included *T. mutans*, *T. buffeli*, *B. occultans* and *Theileria* sp. (sable) in CNP and *A. marginale* subsp. *centrale*, *Theileria* sp. (buffalo) and *A. marginale* in OD. Generally speaking, the buffalo population in the OD sample had lower levels of haemoparasite infections than the one in the CNP sample, with the exception of *Theileria* sp. (buffalo) and to a lesser extent *Anaplasma* sp. Omatjenne and *B. bovis* (in the two later cases, with very few positives were detected). In the specific case of *T. parva,* a significant association was observed between densities of buffalo in the capture location of the herds (only 3 measures of density were available) and prevalence found with the different tests, particularly in the case of the IFAT and qPCR results. This was more evident when comparing those areas where differences between density figures were more extreme (cf MGR and the NG30). However, buffalo density is only one possible cause of those differences, and many other habitat variations or ecological factors affecting host health or vector distribution and density in the different range areas where the buffalo herds were sampled, could also be responsible for those differences [41,42]. Therefore, further studies with a higher number of data and measures at the different herd locations would be necessary to detect explanatory factors accounting for those prevalence differences.

*T. parva* antibodies were detected in 74.1% of samples tested using the IFAT. Limitations of the IFAT include standardization of the test in buffalo samples, subjectivity towards the interpretation of results acquired and the difficulty of detecting low levels of parasite antibodies [9,43]. The IFAT is highly sensitive when testing for antibodies for only one species of *Theileria*, but in areas where different species overlap; cross-reactions between *Theileria* species are common (especially between *T. parva*, *T. annulata* and *T. taurotragi*) and reduce the specificity of the test [9,40]. However, the geographical distribution of *T. annulata* and *T. parva* does not overlap [44] and *T. taurotragi* was not identified in the Botswana buffalo samples. Therefore, the likelihood of cross-reactivity can be ruled out and our results most likely reflect the real presence and burden of *T. parva,* which would explain the high level of agreement between IFAT and qPCR. Another factor to take into account is the period elapsed from infection and the development of antibodies. With the use of schizont antigen, *T. parva* antibodies can first be detected 10 to 14 days post-infection and with piroplasm antigens 15 to 21 days post-infection. High levels of antibodies are still detectable 30 to 60 days after the animals have recovered from a *T. parva* infection which is followed by the gradual decrease of antibody levels. Antibodies can still be detected 4 to 6 months post-recovery and may persist for up to a year at such low levels that they may not be detected at a serum dilution of 1/40 [39]. The animals in our study may have carried *T. parva* infections for a very long time as suggested by the very low antibody levels observed.

Comparing the efficiency of the different tests, we found that the qPCR (80.7%) and IFAT (74.1%) were far better in identifying *T. parva* positive samples than the RLB assay (60.0%). Although there was correlation between the qPCR and RLB results in the detection of *T. parva*, minor differences between the results were observed; most notably in those samples with mixed haemoparasite infection. The qPCR assay can reliably detect *T. parva* in carrier animals with a piroplasm parasitaemia as low as 8.79 × 10^−4^% [16]. The sensitivity of the RLB assay was determined at 10^−6^% parasitaemia, by testing serial dilutions of *T. annulata*-infected blood samples [15]. However, due to the likely presence of multiple parasites present in one sample, competition for available primers may occur in the PCR which in turn would lead to an underrepresentation of some of the parasites detected by RLB alone. Furthermore, weak hybridization and/or cross-reactivity of probes may cause the RLB hybridization assay to yield less sensitive results than the qPCR and IFAT [45]. In addition, mixed infections could mask the presence of novel genotypes in the RLB assay and other tests would be needed to identify them [46,47,15].

Risk factors influencing the prevalence of tick-borne parasites may include the distribution of tick vectors, the abundance of buffalo and cattle and their movement/migratory patterns, resistance of the hosts to the parasites and their tick vectors [48] and age of the host [42]. It has previously been found that older animals have a higher tick load than younger ones [42]. However, higher tick loads do not necessarily mean higher infection rates of haemoparasites. A model designed in East-Africa also determined that in wildlife-livestock interfaces where only cattle were treated with acaricides, *T. parva* remained a problem because this treatment had no effect on the disease transmission in buffalo. In addition, the continuous use of acaricides can have significant economic and ecological consequences [49]. Our study only identified a significant difference between age and infection rate for *B. occultans* and *Theileria* sp. (sable), which depending on the species of parasite, were higher in young (*A. marginale*, *A. marginale* subsp. *centrale*,) or in adult animals (*B. occultans* and *Theileria buffeli*). However, our sample was too small to be able to detect a consistent trend between age and infestation.

A number of studies have previously been conducted on buffalo in South Africa. In the Marakele National Park (MNP) and Kruger National Park (KNP) buffalo were tested for the presence of *Theileria* spp. using the RLB hybridization assay and the *T. parva-*specific qPCR assay [50]. The RLB results indicated the presence of *T. parva*, *Theileria* sp. (buffalo), *T. mutans, T. buffeli* and *T. velifera* in both parks. The qPCR assay identified 70% of samples positive for *T. parva* and the RLB results indicated 40% of samples in both parks. In a separate study in the Hluhluwe-iMfolozi Park and the Greater Limpopo Transfrontier Park, *T. parva*, *Theileria* sp. (buffalo), *T. mutans, T. buffeli* and *T. velifera* were also identified in African buffalo [7]*.* In both studies, the *T. parva*-specific qPCR was found to be more sensitive than the RLB, correlating with the results found in our study. In another study done in the Hluhluwe-iMfolozi Park and Kruger National Park, Debiela [51] found the same *Theileria* spp. as in our study in Botswana, as well as *A. marginale* subsp. *centrale*, *A. marginale*, *Anaplasma* sp. Omatjenne, *E. ruminantium* and *B. occultans*.

In East Africa, in a study done in four different national parks in Uganda, buffalo were found to be carriers of *T. parva, T. mutans, T. velifera, A. marginale* and *A. marginale* subsp. *centrale* [52]. In two of these parks, buffalo also carried *T. buffeli* and *Theileria* sp. (buffalo). None of the animals sampled were carriers of *T. taurotragi, B. bovis, B. bigemina, A. bovis* or *E. ruminantium*. As in Uganda, the pathogenic *B. bovis* has previously been reported to be absent from buffalo in Botswana [53]. However, in the current study, we identified the parasite to be present in a low percentage of the OD buffalo tested. Similarly, *E. ruminantium* could be identified in a few CNP and OD buffalo tested. The significance of buffalo as possible reservoir host of some of these economically important haemoparasites (i.e. *A. marginale, E. ruminantium*) remains unknown.

*Theileria* sp. (sable), which is fatal to sable (*Hippotragus niger*) and roan antelope (*Hippotragus equinus*), but non-pathogenic to buffalo [34] was identified in some of the Botswana buffalo. However, it should be noted that the positive RLB signals might be due to cross reactions of the *Theileria* sp. (sable) probe with genotypes similar to *Theileria* sp. (sable) and/or with *T. velifera* and should be interpreted with caution [12]. Similarly, four samples tested positive for *T. ovis* which is usually found in goats and sheep. We can only speculate whether these are true findings due to incidental infections, or whether they are as a result of cross-reaction of the RLB probes with previously unknown targets or contamination with other target DNA.

The following important tick vectors have been identified in Botswana in previous studies: *Amblyomma variegatum, Rhipicephalus decoloratus, R. zambeziensis, R. evertsi evertsi, R. simus, Hyalomma truncatum* and *H. marginatum rufipes* [54-56]. These ticks are known to transmit most of the haemoparasites found in this study. These vectors may also be responsible for the transmission of *T. buffeli* and *Theileria* sp. (buffalo) but further research is needed to confirm this hypothesis, since the tick vectors of these parasite species remain unknown.

*T. parva* is known to occur in Zambia [8] and South Africa [57,58], but this is the first written report of its occurrence in northern Botswana, despite its presence has been suspected for several years. Currently, there are no regulations instituted for the systematic surveillance and control of tick-borne diseases in Botswana. In addition, Corridor Disease in cattle is fulminant and makes it difficult to detect clinical cases in live animals. Therefore, the present work emphasizes the role of the African buffalo, as a sentinel species to identify the presence and circulation of livestock pathogens. The presence of *Rhipicephalus appendiculatus* and East Coast fever in northern Botswana has been predicted in spatial risk models by some authors [59]. When infected buffalo share the same home ranges with cattle and other domestic animals, those haemoparasites can be transmitted to cattle through infected tick bites. This information on the circulation of TBD can contribute to raise awareness among veterinary officials and rural communities living at the wildlife-livestock interface so that control measures (prevention of wildlife-cattle contacts, regular dipping) can be implemented to mitigate their economic impact. In a recent comparative assessment of cattle herds in three different wildlife/livestock interfaces in Zimbabwe, significantly higher levels of *T. parva* antibodies were found in those areas that were unfenced when compared with those that had a physical separation between wildlife and livestock [60]. Therefore, we can hypothesize that this parasite is less likely to be transmitted from buffalo to cattle in the OD, due to the presence of a veterinary cordon fence preventing contacts with cattle and surrounding the game reserve. To the contrary in the CNP, where there is no physical separation between buffalo and cattle, transmission of common diseases from buffalo to cattle is likely to occur more frequently [29] and future surveillance efforts should be targeted in priority towards livestock from this area.

## Conclusions

This paper illustrates the diversity of haemoparasites present in African buffalo from northern Botswana and highlights the role of African buffalo as a sentinel species for livestock tick-borne pathogens. Our results indicate the significance of the African buffalo as reservoir host for important tick-borne haemoparasites that can cause severe disease in cattle*.* They also suggest that qPCR and IFAT are more efficient in detecting *T. parva* exposed buffalo than the RLB test. These results should contribute to raise awareness among veterinary authorities regarding the potential occurrence of these parasites in cattle so that appropriate control and surveillance protocols taking into account the presence of infected wildlife reservoirs in those areas can be designed at the wildlife-livestock interface.
